# Comparison of graphical optimization or IPSA for improving brachytheraphy plans associated with inadequate target coverage for cervical cancer

**DOI:** 10.1038/s41598-017-16756-w

**Published:** 2017-11-27

**Authors:** ZhiJie Liu, HuanQing Liang, Xiao Wang, HaiMing Yang, Ye Deng, TingJun Luo, ChaoFeng Yang, Min Lu, QingGuo Fu, XiaoDong Zhu

**Affiliations:** 1 Department of Radiation Oncology, Cancer Institute of Guangxi Zhuang Autonomous Region, The Affiliated Cancer Hospital of Guangxi Medical University, Nanning, 530021 PR China; 2 Department of Radiation Oncology, Nanfang Hospital, Southern Medical University, Guangzhou, 510515 PR China; 3 Department of Radiation Oncology, Rutgers Cancer Institute of New Jersey, New Brunswick, NJ 08903 USA

## Abstract

Many studies have reported that inverse planning by simulated annealing (IPSA) can improve the quality of brachytherapy plans, and we wanted to examine whether IPSA could improve cervical cancer brachytherapy plans giving D_90_ < 6 Gy (with 7 Gy per fraction) at our institution. Various IPSA plans involving the tandem and ovoid applicators were developed for 30 consecutive cervical cancer patients on the basis of computed tomography: IPSA1, with a constraint on the maximum dose in the target volume; IPSA1-0, identical to IPSA1 but without a dwell-time deviation constraint; IPSA2, without a constraint on the maximum dose; and IPSA2-0, identical to IPSA2 but without a dwell-time deviation constraint. IPSA2 achieved similar results as graphical optimization, and none of the other IPSA plans was significantly better than graphical optimization. Therefore, other approaches, such as combining interstitial and intracavitary brachytherapy, may be more appropriate for improving the quality of brachytherapy plans associated with inadequate target coverage.

## Introduction

Cervical cancer is very common in China, and brachytherapy plays an important role in its treatment^1^. Brachytherapy plans were historically developed using radiographs and point dosimetry systems^2^, and today 3-D image-guided brachytherapy is used to optimize the dose distribution to the target volume and avoid high doses to organs at risk^3–5^. Inverse planning by simulated annealing (IPSA) can allow the use of lower radiation doses while maintaining or improving target coverage when planning brachytherapy involving tandem and ovoid applicators in cervical cancer^6^. Other studies have reported similar results when comparing IPSA with dose point optimization, manual optimization of dwell weights/times and geometric optimization for planning brachytherapy involving a tandem and ovoid^7–11^.

In our hospital, some brachytherapy plans based on the graphical optimization (GrO) algorithm do not give adequate target coverage (providing D_90_ < 6 Gy and 7 Gy per fraction) when the dose to organs at risk is kept within recommended limits routinely used at our institution. Here we investigated whether such plans could be improved using IPSA. Our primary criterion for improvement was D_90_, and secondary criteria were V_150_, V_200_ and dwell time.

## Results

### Comparison of IPSA1, IPSA2 and GrO

**Figure 1:**
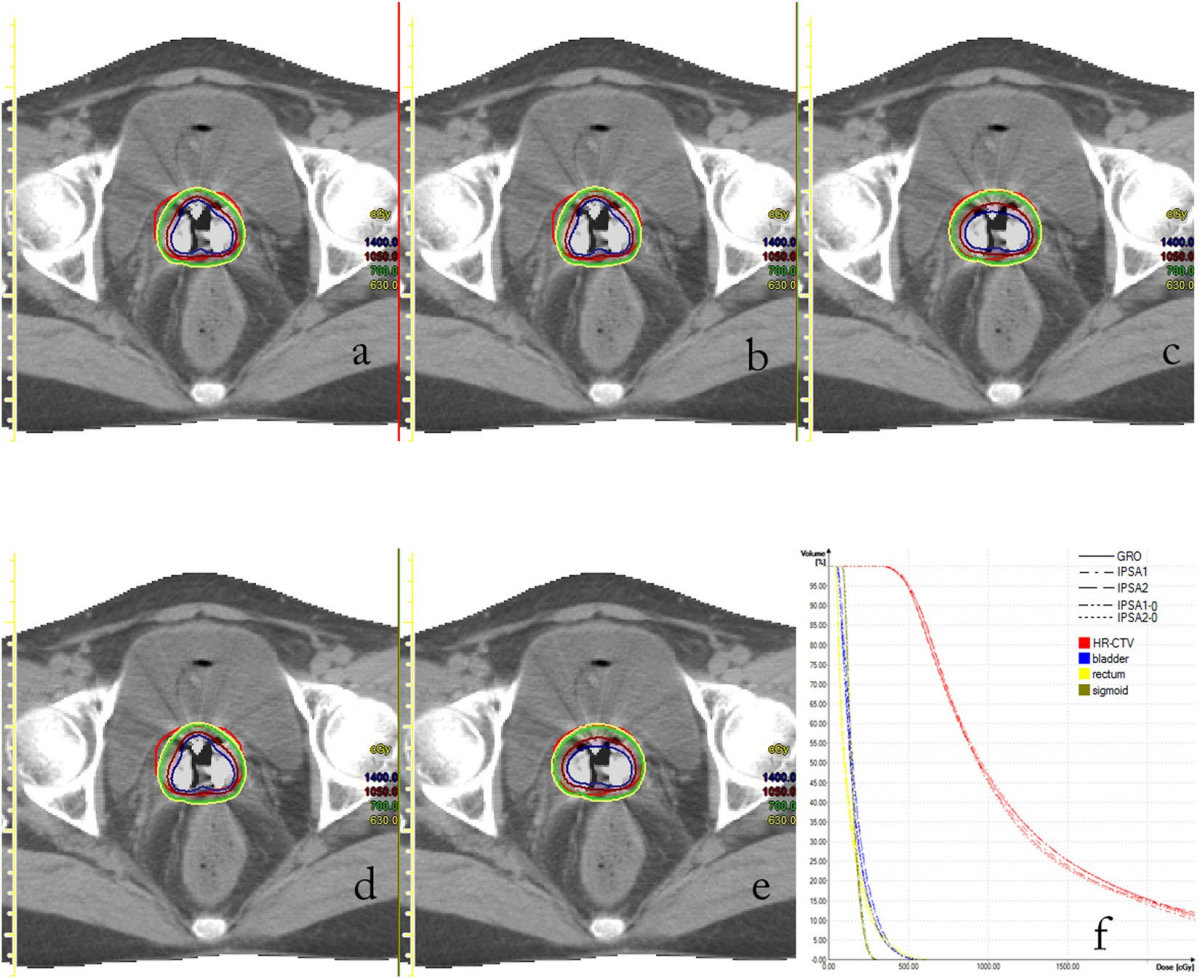
Example of transverse computed tomography images and dose-volume histogram from one patient for brachytherapy planned using (**a**) GrO, (**b**) IPSA1, (**c**) IPSA2, (**d**) IPSA1-0, or (**e**) IPSA2-0. (**f**) The dose-volume histogram.

Mean D_90_ was 0.2 Gy smaller with IPSA1 than with GrO (3.8%, *p* = 0.031; Table 1 and Fig. 1), while D_90_ was 0.4 Gy higher with IPSA2 than with IPSA1 (8.0%, *p* = 0.002), illustrating the effect of removing the V_max_ restriction. Of the three planning algorithms, IPSA1 had the smallest V_150_ and V_200_ (*p* = 0.036 and 0.030 *vs*. GrO; *p* = 0.037 and 0.032 *vs*. IPSA2).

**Table 1:** Comparison of results with each plan.

parameters	GrO	IPSA1	IPSA2	IPSA1-0	IPSA2-0	p value
D100(Gy)	3.2 ± 0.5	3.1 ± 0.6	3.5 ± 0.5^a,c,d^	3.4 ± 0.5^a,c,d^	3.6 ± 0.5^a,b,c^	0.042
D90(Gy)	5.2 ± 0.6	5.0 ± 0.6^e^	5.4 ± 0.6^a^	5.2 ± 0.6^b,d^	5.4 ± 0.6^a^	0.038
V100(%)	66.2 ± 8.8	62.7 ± 9.7^e^	68.0 ± 9.4^a^	65.9 ± 8.8^b,d^	68.8 ± 9.2^a^	0.036
V150(%)	35.9 ± 5.8	33.4 ± 6.7^e^	35.8 ± 5.6	34.7 ± 5.5^e^	36.2 ± 5.6	0.022
V200(%)	21.9 ± 3.5	19.6 ± 3.7^e^	21.4 ± 3.4	20.7 ± 3.5^e^	21.7 ± 3.4	0.041
CNI	0.62 ± 0.08^e^	0.57 ± 0.09^e^	0.59 ± 0.09	0.58 ± 0.08	0.60 ± 0.09	0.042
CI	0.94 ± 0.06^e^	0.91 ± 0.04^e^	0.87 ± 0.06	0.89 ± 0.04^e^	0.87 ± 0.06	0.016
Bladder2cc(Gy)	4.4 ± 0.1^e^	4.5 ± 0.03	4.5 ± 0.07	4.5 ± 0.04	4.5 ± 0.08	0.027
Rectum2cc(Gy)	3.6 ± 0.4	3.4 ± 0.4^e^	3.5 ± 0.4	3.5 ± 0.4	3.6 ± 0.4	0.044
Sigmoid2cc(Gy)	2.7 ± 1.0	2.5 ± 0.9	2.7 ± 1.0	2.7 ± 1.0	2.8 ± 1.0	0.653
Total dwell time(s)	587.1 ± 304.9^e^	565.1 ± 289.4^e^	632.0 ± 324.8	602.7 ± 305.5^e^	643.0 ± 330.3	0.002
Max dwell time(s)	54.2 ± 66.9	50.8 ± 35.5	68.0 ± 35.5	111.8 ± 75.4^e^	74.2 ± 53.1	0.000
SD of dwell time (s)	12.6 ± 13.7	13.3 ± 9.2	18.0 ± 15.3^a,c^	22.8 ± 14.7^e^	19.0 ± 13.2^a,c^	0.003

Results are mean ± 1 standard deviation.

^a^Significantly different from the GrO.

^b^Significantly different from the IPSA2.

^c^Significantly different from the IPSA1.

^d^Significantly different from the IPSA2-0.

^e^Significantly different from all other plans.

**Figure 2:**
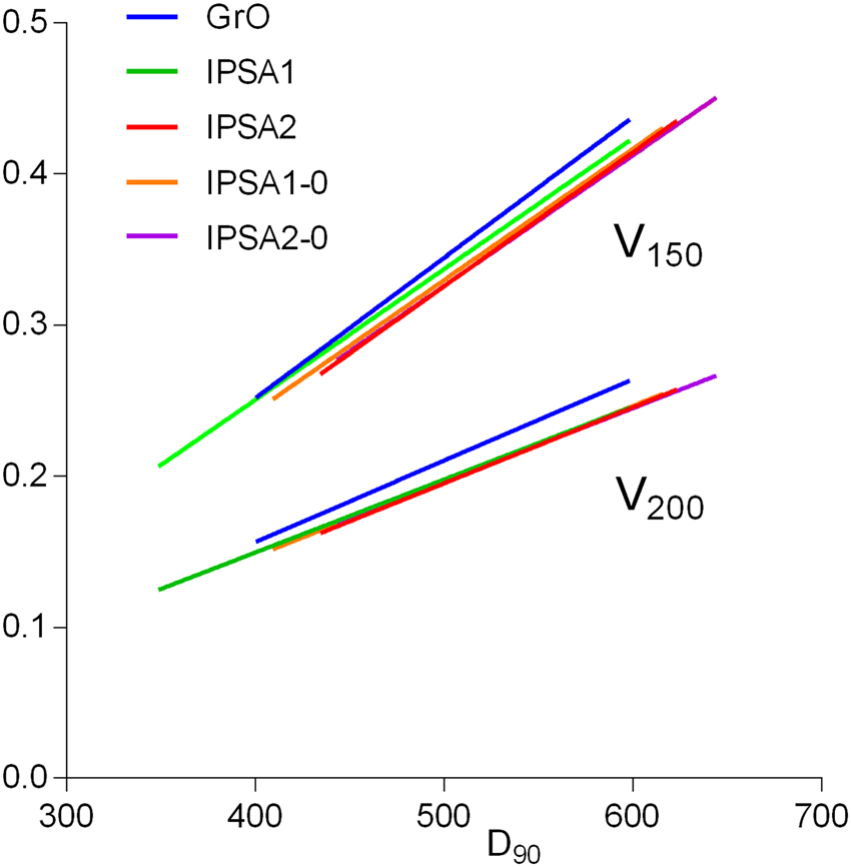
Linear relationship among D_90_, V_150_ and V_200_.

A direct linear relationship was observed between high-risk clinical target volume (HR-CTV) D_90_ and high dose in the target (Fig. 2). The IPSA2 line lies beneath the two straight lines of IPSA1 and GrO indicating that IPSA2 may be associated with smaller V_150_ than IPSA1 or GrO for the same HR-CTV D_90_. The length of each line represents the range of the HR-CTV D_90_, and a considerable part of the line in the case of IPSA1 is biased towards the left side of the *x* axis, indicating that it provided the smallest average high-dose volume. Lines for the various IPSA algorithms nearly coincide for V_200_.

**Figure 3:**
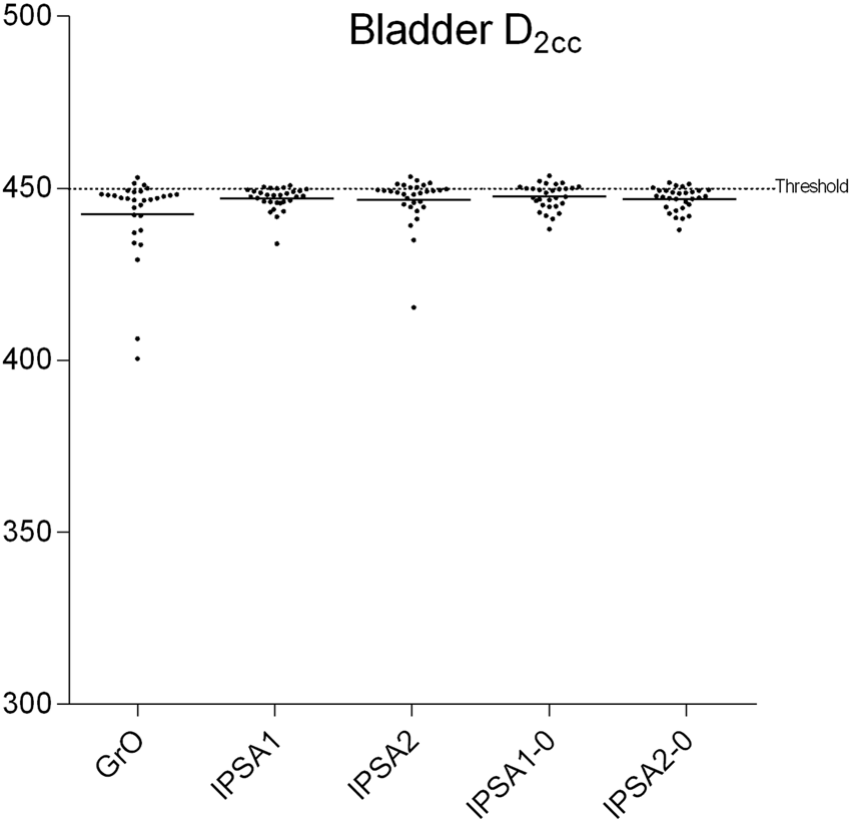
Comparison of bladder D_2cc_ among different plans.

**Figure 4:**
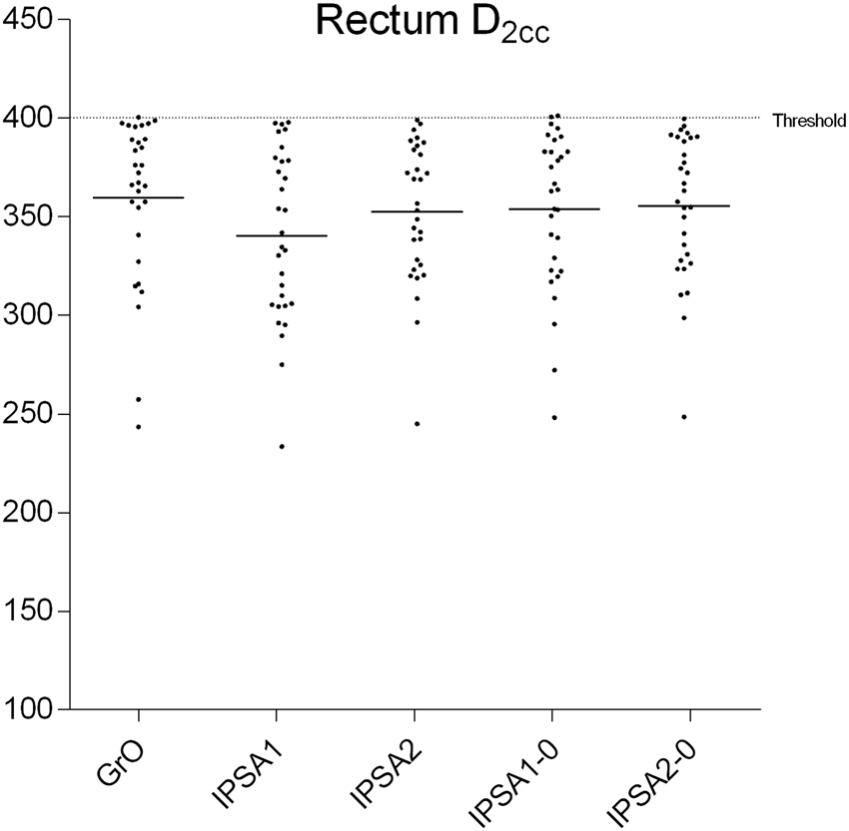
Comparison of rectum D_2cc_ among different plans.

**Figure 5:**
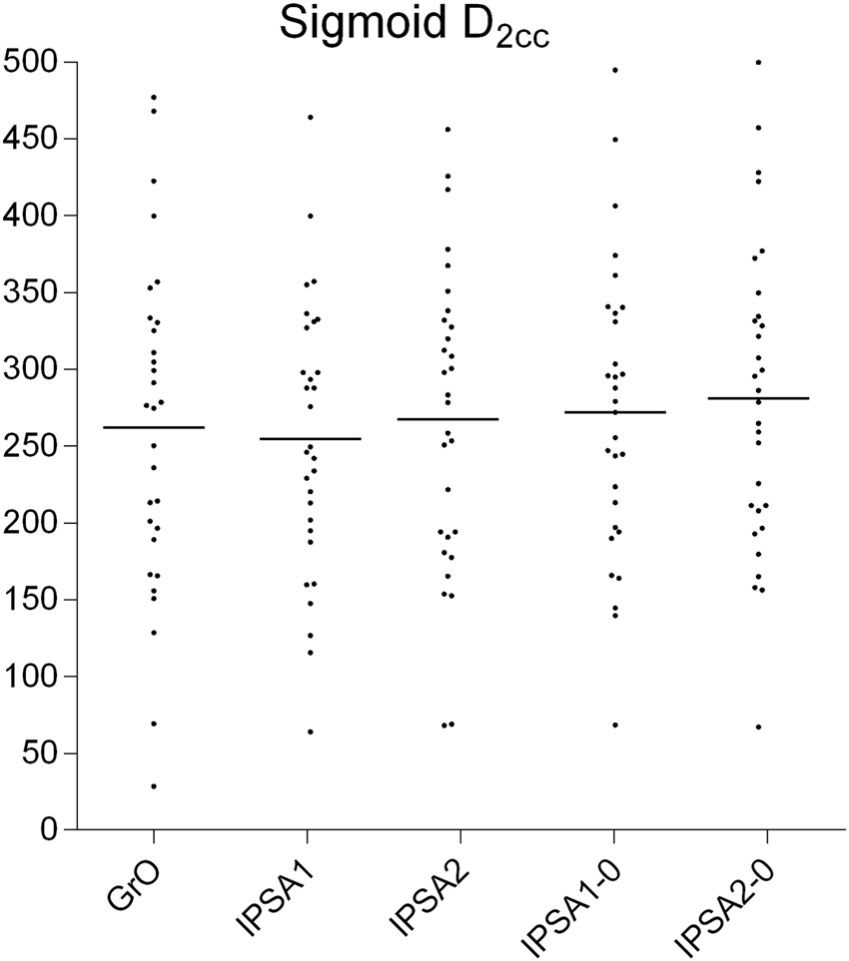
Comparison of sigmoid D_2cc_ among different plans.

Figures 3–5 show that the concentration of D_2cc_ points near the dose limit is greater at the bladder than at other organs at risk. This indicates that all the plans tested expose the bladder to a similar dose, implying that the bladder may be the organ at risk that most limits target coverage. GrO offered significantly lower dose to the bladder than the IPSA plans (*p* = 0.012), whereas IPSA1 offered significantly lower dose to the rectum (*p* = 0.001).

The conformity index (CI) of GrO was 0.94; IPSA1, 0.91; and IPSA2, 0.87 (*p* = 0.002; Table 1). The corresponding values of the conformal index (COIN) were 0.62, 0.57 and 0.59 (*p* = 0.000). There was no significant difference in maximum dwell time among the three plans (*p* = 0.526), although IPSA2 showed significantly longer total dwell time and greater dwell-time standard deviation than the two other methods (*p* = 0.002, *p* = 0.003).

### Comparing IPSA plans with and without dwell-time deviation constraint

There was significant difference in D_90_ between IPSA1 and IPSA1-0 (*p* = 0.047) while there was no significant difference between IPSA2 and IPSA2-0 (*p* = 0.781). The V_150_ and V_200_ of IPSA1-0 were significantly higher than those of IPSA1 (34.7% *vs* 33.4%, *p* = 0.001; 20.7% *vs* 19.6%, *p* = 0.013). COIN and CI were also significantly different between IPSA1-0 and IPSA1 (*p* = 0.026, *p* = 0.001).

Total dwell time, maximum dwell time and dwell-time standard deviation were significantly larger with IPSA1-0 than with IPSA1 (*p* = 0.003, *p* = 0.000, *p* = 0.000), reflecting the lack of a constraint on dwell-time deviation. These three parameters did not differ significantly between IPSA2-0 and IPSA2 (*p* = 0.642, *p* = 0.574, *p* = 0.663).

## Discussion

The IPSA1 plan did not provide better target coverage than GrO. The IPSA2 plan improved D_90_ by 0.4 Gy (8%) relative to IPSA1, reflecting the effect of removing the V_max_ restriction; however, this improved D_90_ by only 0.2 Gy relative to GrO. Further removing the dwell-time deviation constraint led to increases in V_150_, V_200_, total dwell time, maximum dwell time and dwell-time standard deviation.

We found a linear relationship between D_90_ and high dose at the target (Fig. 2). This likely reflects a fundamental limitation of the tandem and ovoid applicators: since only one catheter lies within the uterine cavity, the isodose lines form concentric circles around it, reflecting the fact that dose falls off by the inverse square law. As a result, achieving the prescribed dose on the surface of a large target necessarily implies a large high-dose volume at the target.

The optimization parameter V_max_ in IPSA1 may not be the most effective guide for brachytherapy planning, since it operates in opposition to the “minimum surface dose” parameter. Dose distribution is quite sensitive to V_max_, which can decrease dose to organs at risk, high-dose volume in the target as well as target coverage^12^. We found that removing the V_max_ constraint improved target coverage while keeping the dose to organs at risk within limits. Thus, IPSA2 showed greater V_150_ and V_200_ than IPSA1 while keeping the high-dose volume similar to that with GrO. These results suggest that the “maximum surface dose” parameter used in IPSA2 may be more reasonable and effective.

At the same time, removing the V_max_ restraint increased target coverage by 0.2 Gy relative to GrO, which does not substantially improve plan quality. In fact, the various IPSA plans succeeded only in optimizing dwell time and dwell position relative to GrO. In all plans, a large part of the target lies close to the bladder or rectum, or the target extends laterally into the region in the case of a tandem applicator. This suggests that dose distribution is affected mainly by the location of the target and organs at risk, the shape of the target and the placement of the catheters. If all these factors are fixed, optimization of dwell time and dwell position can, at best, merely fine-tune the dose distribution. These findings are consistent with other studies showing that IPSA can minimize dose to organs at risk and maximize target coverage without substantially altering the dose distribution in brachytherapy for prostate cancer^13,14^ or gynecologic cancers^7,8,10^.

Using different applicators may substantially improve plan quality. In contrast to our increase of 0.2 Gy in mean D_90_ achieved through plan optimization, the combination of interstitial and intracavitary brachytherapy can increase D_90_ by about 1 Gy (17.5%) relative to intracavitary brachytherapy alone^11^. Similarly, using Vienna applicators rather than tandem and ovoid applicators increased D_90_ by 1.7 Gy (27.9%)^9^, and interstitial brachytherapy has been shown to give higher mean D_90_ than intracavitary brachytherapy^15^.

We found that removing the dwell-time deviation constraint from IPSA1 and IPSA2 increased target coverage and dwell-time deviation, consistent with a previous report^16^. However, the change in D_90_ was relatively small in our study. In addition, the increase in dwell-time deviation can generate isolated dwell positions with long dwell times, which may increase the risk of hot spots. These hot spots may migrate onto organs at risk if the catheter shifts after computed tomography. Therefore, removing the dwell-time deviation constraint may not be an appropriate method for improving target coverage.

IPSA2 significantly increased target coverage without increasing V_150_ or V_200_ relative to GrO. At the same time, IPSA2 showed larger dwell-time deviation than GrO and IPSA1. Increasing the dwell-time deviation constraint may reduce dwell-time deviation in IPSA2^16^.

Although our results reflect the particular approach for target delineation and plan evaluation in place at our hospital, our findings may be useful for brachytherapy planning at other institutions. We found that none of the IPSA plans substantially improved brachytherapy quality above GrO, and the IPSA2 plan achieved similar results as GrO. The “maximum surface dose” parameter may be more reasonable and effective for decreasing high dose volume of brachytherapy plans giving poor performance (D_90 < _6 Gy with 7 Gy per fraction) using tandem and ovoid applicators. Removing the dwell-time deviation constraint may increase the risk of harm to normal tissue without improving target coverage.

## Methods

### Patients

Computed tomography images were used to re-plan the brachytherapy treatment plans based on tandem and ovoid applicators for 30 consecutive patients (mean age, 48 yr) with cervical cancer in stage IIB-IIIB based on the International Federation of Gynecology and Obstetrics staging system. These patients were treated at the Affiliated Cancer Hospital of Guangxi Medical University between December 2015 and June 2016. Patients were treated with external beam radiation therapy of 50 Gy in 25 fractions to the target and were concurrently given chemotherapy. This combination therapy was followed by high-dose-rate brachytherapy of 28 Gy in 4–5 fractions.

This study was approved by the Ethics Committee at the Affiliated Cancer Hospital of Guangxi Medical University. All procedures were in accordance with national and international ethical guidelines. Informed consent was obtained from all participants.

### Contouring

Each brachytherapy fraction was followed by computed tomography scanning and contour delineation. Therefore, every patient had four or five computed tomography image sets and contours of the region of interest. Only one fraction from each patient was used in this study. HR-CTVs and organs at risk (rectum, bladder and sigmoid)^17^ were delineated by a gynecologist.

Contouring and treatment planning were performed using Oncentra^®^ Brachy software (version 4.3, Elekta). One GrO plan and four IPSA plans (see below) were prepared from each computed tomography image set. HR-CTV per fraction was defined to be 7 Gy, maximum bladder dose could not exceed 4.5 Gy, and maximum rectum dose could not exceed 4 Gy. In some cases, limiting the dose to organs at risk was given higher priority than target coverage. This was decided by the physician based on prognostic factors and institutional procedures. When target coverage was insufficient, an additional fraction of high-dose-rate brachytherapy was delivered in order to cover the target with an equivalent dose in 2 Gy-fractions (EQD2) of 80–90 Gy.

### Treatment planning

#### GrO plans

GrO plans were first optimized via dose points optimization. After digitizing applicators, the dwell positions, separated by a 2.5-mm step size, were determined by the extent of the HR-CTV. Dose distributions were then dose-points-optimized with 300 target points randomly placed at the surface of the HR-CTV. After dose points optimization, the GrO was applied to adjust isodose lines using the mouse to achieve the desired target coverage while keeping the doses to organs at risk below the given constraints. The GrO plans were used in actual treatments.

#### IPSA plans

Two-class solutions shown in Table 2 were used as starting points for IPSA1 and IPSA2. The main differences between IPSA1 and IPSA2 were that IPSA1 had a constraint on the maximum dose to the target volume (V_max_), and IPSA1 assigned a slightly higher weight to the “minimum surface dose” parameter. The dwell-time deviation constraint was set to 0.2 in both IPSA1 and IPSA2. To further improve target coverage, this constraint was set to 0 in these plans to generate the respective plans IPSA1-0 and IPSA2–0. The organs at risk were set to the same value in the two-class solutions. After running the optimization with the class solution, dose objectives and weighting factors were modified for individual patients when necessary in order to optimize the dose distribution. After the final results were obtained in the IPSA plans, no fine-tuning of the dose distribution using GrO was allowed.

**Table 2:** Dose objectives and weighting factors used for IPSA plans.

	Plan	Region of interest	Surface dose				Volume dose			
			Weight	Min dose	Max dose	Weight	Weight	Min dose	Max dose	Weight
Target	IPSA1	HR-CTV	170	7.0 Gy	7.5 Gy	100	100	7.2 Gy	80.0 Gy	5
	IPSA2	HR-CTV	150	7.0 Gy	7.5 Gy	100	100	7.2 Gy		
Organs at risk		Bladder			4.0 Gy	100				
		Rectum			3.5 Gy	50				
		Sigmoid			4.0 Gy	30				

### Plan evaluation

The following dosimetric parameters of different plans were analyzed based on the dose-volume histograms: HR-CTV D_90_, the dose that covered 90% of the HR-CTV; D_100_, the dose that covered 100% of the HR-CTV; V_100_, the percentage volume covered by at least 100% of the prescribed dose; V_150_, the volume that received at least 150% of the prescribed dose; V_200_, the volume that received at least 200% of the prescribed dose; and D_2cc_, dose covering at least 2 cm^3^ of organs at risk. In addition, COIN and CI^7^, were compared (Eqs 1–2).

Since we were concerned about potential hot spots, we also evaluated the differences in dwell time distributions by recording the mean and maximum dwell time and the mean standard deviation (SD) of the dwell time in each plan.

### Statistical analysis

Differences in the means of each dose parameter among the five plans (GrO, IPSA1, IPSA2, IPSA1-0, IPSA2-0) were assessed for significance using matched ANOVA. Two-group comparisons were assessed for significance using the least-squares difference test. Two-tailed values of P < 0.05 were considered statistically significant. All data analyses were performed using SPSS (IBM, Chicago, IL, USA) and GraphPad Prism 5 (Graphpad, USA).

### Data availability

The datasets analyzed in the present study are available from the corresponding author upon reasonable request.

1 $${\rm{COIN}}={{\rm{CTV}}}_{{\rm{target}}}\times {{\rm{CTV}}}_{{\rm{target}}}{/({\rm{V}}}_{{\rm{CTV}}}\times {{\rm{V}}}_{{\rm{total}}})$$

2 $${\rm{CI}}={{\rm{CTV}}}_{{\rm{target}}}/{{\rm{V}}}_{{\rm{total}}}$$

CTV_target_ is the part of the HR-CTV receiving at least the prescribed dose, V_total_ is the total volume receiving at least the prescribed dose, and V_CTV_ is the volume of the HR-CTV.
